# Permafrost microbial communities and functional genes are structured by latitudinal and soil geochemical gradients

**DOI:** 10.1038/s41396-023-01429-6

**Published:** 2023-05-22

**Authors:** Mark P. Waldrop, Christopher L. Chabot, Susanne Liebner, Stine Holm, Michael W. Snyder, Megan Dillon, Steven R. Dudgeon, Thomas A. Douglas, Mary-Cathrine Leewis, Katey M. Walter Anthony, Jack W. McFarland, Christopher D. Arp, Allen C. Bondurant, Neslihan Taş, Rachel Mackelprang

**Affiliations:** 1 Geology, Minerals, Energy, and Geophysics Science Center, United States Geological Survey, Menlo Park, CA 94025 USA; 2 California State University Northridge, 18111 Nordhoff St., Northridge, CA 91330 USA; 3 GFZ German Research Centre for Geosciences, Section Geomicrobiology, 14473 Potsdam, Germany; 4 University of Potsdam, Institute of Biochemistry and Biology, 14476 Potsdam, Germany; 5 Earth and Environmental Sciences Area, Lawrence Berkeley National Laboratory, Berkeley, CA USA; 6 U.S. Army Cold Regions Research and Engineering Laboratory 9th Avenue, Building 4070 Fort, Wainwright, AK 99703 USA; 7 Agriculture and Agri-Food Canada, 2560 Boulevard Hochelaga, Québec, QC G1V 2J3 Canada; 8 Water and Environmental Research Center, University Alaska Fairbanks, Fairbanks, AK 99775 USA

**Keywords:** Biogeochemistry, Soil microbiology

## Abstract

Permafrost underlies approximately one quarter of Northern Hemisphere terrestrial surfaces and contains 25–50% of the global soil carbon (C) pool. Permafrost soils and the C stocks within are vulnerable to ongoing and future projected climate warming. The biogeography of microbial communities inhabiting permafrost has not been examined beyond a small number of sites focused on local-scale variation. Permafrost is different from other soils. Perennially frozen conditions in permafrost dictate that microbial communities do not turn over quickly, thus possibly providing strong linkages to past environments. Thus, the factors structuring the composition and function of microbial communities may differ from patterns observed in other terrestrial environments. Here, we analyzed 133 permafrost metagenomes from North America, Europe, and Asia. Permafrost biodiversity and taxonomic distribution varied in relation to pH, latitude and soil depth. The distribution of genes differed by latitude, soil depth, age, and pH. Genes that were the most highly variable across all sites were associated with energy metabolism and C-assimilation. Specifically, methanogenesis, fermentation, nitrate reduction, and replenishment of citric acid cycle intermediates. This suggests that adaptations to energy acquisition and substrate availability are among some of the strongest selective pressures shaping permafrost microbial communities. The spatial variation in metabolic potential has primed communities for specific biogeochemical processes as soils thaw due to climate change, which could cause regional- to global- scale variation in C and nitrogen processing and greenhouse gas emissions.

## Introduction

Permafrost is a crucial part of ecosystem biogeochemistry in high latitude soils. The microbial communities within permafrost slowly transform organic matter though geologic time, shaping the permafrost environment, and contribute to greenhouse gas emissions upon thaw. Microbial communities vary over space and time and are strongly affected by ecosystem and soil characteristics such as pH, vegetation, temperature, and precipitation across broad environmental gradients [1–3]. Yet in permafrost, microbial communities are often disconnected from modern ecosystems, drawn from communities that were present prior to permafrost formation and modified over time [4, 5].

Permafrost has formed at multiple times in the past, and its presence is dependent on geography and local site conditions. Much of the permafrost in northern latitude soils was formed during the late Pleistocene (11,700–129,000 years) [6]. While Holocene (present – 11,700 years) aged permafrost can be hundreds to thousands of years old, rates of formation were greatest during the Little Ice Age (~1300 to 1850 CE) [7]. Permafrost soils may be derived from wetlands, glaciofluvial deposits, loess, or weathered bedrock [8]. Due to differences in glaciation, loess deposition rates, and thaw events driven by disturbances such as wildfire, there is often a strong landscape-scale variation in permafrost age, mode of formation, and physical structure that is likely reflected in the microbial communities that reside there.

Permafrost microbial communities are influenced by the age of permafrost aggradation [9], sedimentation, thaw history, marine exposure [10, 11], permafrost origin [12], and mineral composition [13]. Prominent members of the permafrost community include *Proteobacteria* and *Actinobacteria* whereas *Firmicutes, Bacteroidetes*, and *Chloroflexi* can vary substantially with age and other physicochemical factors [9, 14]. Most permafrost Archaea belong to methanogens from *Euryarchaeota* [14], though recent studies have identified *Thaumarchaeota*, *Bathyarchaeota*, and *Heimdallarchaeota* [10, 11, 15]. Consequently, permafrost microbial communities typically reflect local climates, vegetation, and soil properties present at the time of formation. But over geologic time (with little influx of water, resources, or microbial immigrants), diversity declines and genetic potential changes [4, 5, 14]. Although we know something about the composition and diversity of permafrost microbial communities, we know much less about the genetic composition that gives rise to community function in situ.

**Fig. 1:**
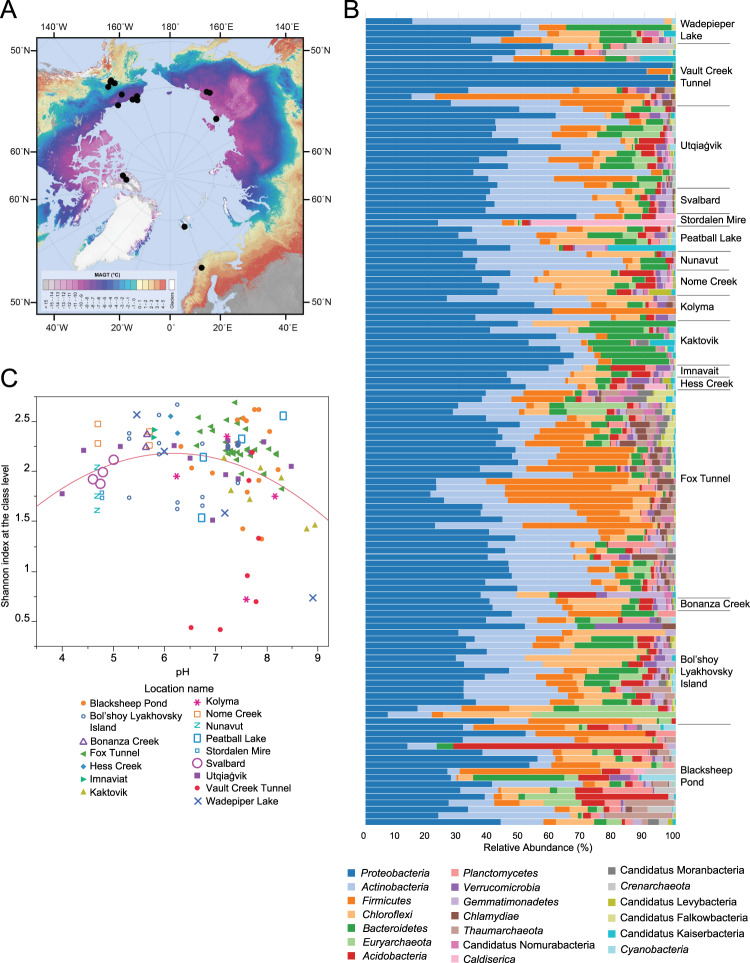
Geographic distribution of sampling locations and taxonomic composition of microbial communities in permafrost. **A** Permafrost sample locations overlayed onto a map showing mean annual ground temperature (MAGT) [46]. Map used with permission. **B** Relative abundance of bacterial and archaeal phyla across permafrost samples. **C** Unimodal relationship between Shannon diversity and permafrost pH. Different colors/shapes represent different locations. *R*^2^ = 0.06, *p* = 0.019. Shannon diversity at the class level = 2.945 - 0.116*pH - 0.0668*(pH-6.933)^2^. With Vault Creek Tunnel outlier samples excluded *R*^2^ = 0.14, *p* < 0.0001.

Examining large scale biogeography of microbial communities allows us to understand features common to all permafrost communities, as well as factors that vary most among communities. We analyzed 133 permafrost metagenomes from the panarctic, including the United States, Canada, Russia, Sweden, and Svalbard (Fig. 1A, Table S1, Table S2) to (1) identify the dominant permafrost taxa, (2) ascertain the environmental variables shaping the occurrence of taxa and potential functions defined by the relative abundance of functional genes, and (3) identify highly variable genes that reflect selective pressures driving differences among permafrost microbial communities. We determined the distribution of taxa and functional genes across the Arctic and we related them to environmental metadata derived from landform, landform history, and soil chemical attributes. This study provides unprecedented insights into factors structuring microbial communities and their functional potential in this climate-critical ecosystem.

## Materials and methods

### Sample overview

We analyzed 133 permafrost metagenomes from samples collected across the panarctic, including the United States, Canada, Russia, Sweden, and Svalbard (Fig. 1A, Table 1). We sequenced 91 samples and combined these results with data from 42 samples obtained from previously published studies (Tables S1 and S2) [9, 10, 16–25]. Permafrost soils were collected in different ways depending on their depth and access limitations. Within a few meters of the surface, we used a Snow, Ice and Permafrost Research Establishment (SIPRE) core barrel (7.62-cm inner diameter, up to 120 cm in length [26]). For samples tens of meters deep we used a truck-mounted deep core auger which operated without drilling fluid [27]. When we had access to permafrost from tunnels or cliff faces, we collected samples from the sidewalls using a large hole saw attached to a hand drill. Where possible, at least three replicates were collected per site. In all cases, methods were used to ensure soils were free from surface microbial contamination as described previously [9, 17]. Details specific to each location sampled are contained in the Supplementary Information.

**Table 1:** Biophysical properties of permafrost sampling locations (mean ± SD).

*Name*	*n*	*Lat*	*Long*	*Age (kyr)*	*Depth (m)*	*Organic C (%)*	*Nitrogen (%)*	*CN ratio*	*pH*	*Ice content (g /g soil)*
Atqasuk	2	70.472	−157.412	9	0.4	2.1 ± 0.4	0.42 ± 0.14	5.6 ± 2.9	5.56 ± 0.25	0.59 ± 0.13
Blacksheep Pond										
Lake sediment	6	64.888	−147.920	5	Multiple	0.9 ± 0.29	0.05 ± 0.02	16.9 ± 6.5	7.28 ± 0.22	0.24 ± 0.09
Yedoma	9	64.890	−147.916	12	Multiple	1.8 ± 0.84	0.13 ± 0.07	14.9 ± 3.3	7.34 ± 0.67	0.41 ± 0.15
Bol’shoy Lyakhovsky Island^a^										
Holocene^a^	3	73.350	141.242	3	2	0.21	0.11	1.9	5.31	0.14
Pleistocene	9	73.336	141.328	30–54	7	3.57 ± 1.8	0.36 ± 0.07	9.13 ± 3.49	6.29 ± 0.37	0.55 ± 0.23
Eemian	6	73.341	141.286	115–125	5.5	0.59 ± 0.05	0.16 ± 0.03	3.85 ± 1.15	7.33 ± 0.10	0.45 ± 0.21
Bonanza^a^	2	64.760	−148.266	2	0.7	41.0	2.0	20.3	5.76	0.58
Eureka	1	80.000	−85.839	6	2.0	1.4	0.1	14	7.8	0.27
Fox Tunnel										
19 kyr	12	64.952	−147.621	19	12.4	1.97 ± 0.36	0.17 ± 0.03	11.48 ± 0.43	7.68 ± 0.43	0.39 ± 0.02
27 kyr	9	64.952	−147.621	27	14.6	2.85 ± 0.14	0.26 ± 0.02	11.44 ± 0.82	7.59 ± 0.06	1.12 ± 0.10
33 kyr	11	64.952	−147.621	33	15.5	2.78 ± 0.07	0.26 ± 0.01	11.12 ± 0.61	7.07 ± 0.10	0.57 ± 0.04
Hess Creek	2	65.700	−149.082	1	0.8	37.1 ± 4.2	1.63 ± 0.23	22.8 ± 0.7	6.19 ± 0.09	0.48 ± 0.55
Imnavait	2	68.614	−149.314	70	1.0	ND	ND	ND	5.8	ND
Kaktovik	7	70.133	−143.688	13	1.5	2.4 ± 2.2	0.17 ± 0.16	20.2 ± 23.0	8 ± 0.69	1.73 ± 1.44
Kolyma	4	69.252	155.598	10–880	12.9	1.0 ± 0.1	0.13 ± 0.01	7.6 ± 0.2	7.29 ± 0.82	0.23 ± 0.07
Nome Creek	4	65.599	−145.220	10	0.7	5.7 ± 1.8	0.25 ± 0.05	22.3 ± 2.0	5.2 ± 0.57	0.55 ± 0.07
Nunavut^a^	3	79.246	−91.097	6	0.8	1	0.1	10	4.69	0.2
Peatball Lake	4	70.751	−153.869	8	1.6	4.6 ± 1.4	0.31 ± 0.16	16.2 ± 5.1	7.51 ± 0.8	2.55 ± 2.3
Stordalen Mire	4	68.353	19.047	2	0.7	21.0 ± 9.7	1.23 ± 0.4	17.1 ± 4. 5	4.77	ND
Svalbard	4	78.186	15.925	3	1.4	1.91 ± 0.02	2.96 ± 0.75	6.9 ± 1.7	4.78 ± 0.16	0.25 ± 0.04
Utqiaġvik	13	71.323	−156.611	11	1.7	14.9 ± 17.4	1.01 ± 0.92	16.5 ± 7.3	6.66 ± 1.37	0.46 ± 0.2
Vault Creek Tunnel										
25 kyr	3	65.029	−147.699	25	5	2.57 ± 0.27	0.24 ± 0.03	10.77 ± 0.17	6.72 ± 0.03	0.58 ± 0.04
44 kyr	3	65.029	−147.699	44	12	7.29 ± 1.05	0.53 ± 0.08	13.7 ± 1.5	7.08 ± 0.32	1.7 ± 0.25
50 kyr	3	65.029	−147.699	50	20	2.61 ± 0.54	0.19 ± 0.02	13.47 ± 1.19	7.82 ± 0.03	0.4 ± 0.07
90 kyr	2	65.029	−147.699	90	40	4.68 ± 3.96	0.18 ± 0.10	20.5 ± 11.5	7.68 ± 0.03	0.63 ± 0.09
Wadepiper Lake	4	70.710	−153.938	10	1.3	4.7 ± 2.9	0.32 ± 0.21	15.3 ± 4.6	6.88 ± 1.52	ND

Measurements for each sample can be found at ScienceBase.

*n* is indicative of the number of field replicates used for DNA analyses, not the number of replicates for chemical analyses.

*ND* not determined.

^a^Replicate biophysical measurements were not taken or were not available.

### Permafrost age dating, chemical analyses, and core processing

We collected geographic and geomorphic data for each site using their GPS locations. These data included continental region (Siberia, Interior Alaska, Alaskan Tundra, High Arctic Canada, Spitzbergen, and Sweden), permafrost type (continuous versus discontinuous and yedoma versus non-yedoma [28, 29], origin (lacustrine, aeolian, alluvium), and elevation. Permafrost age (e.g., Holocene versus Pleistocene or kya) was derived from existing data or inferred from published literature (Table S2). Soil chemical attributes were collected from existing data or measured as needed (Table 1).

### Core sub-sectioning, DNA extraction, library preparation, and sequencing

Prior to DNA extraction, the outer 1-2 cm of permafrost cores were removed using sterile tools to expose the uncontaminated interior. Cores were further subsectioned with sterile knives and chisels and DNA was extracted from 0.5 g to 2 g using the FastDNA SPIN Kit for soil (MP Biomedicals, Santa Ana, CA, USA) or the DNeasy PowerSoil Kit, Qiagen, Germantown, MD, USA). Sequencing libraries were prepared using either emulsion PCR [9] or the Illumina TruSeq DNA sample preparation kit v2 following the low-throughput protocol and sequenced on a HiSeq 4000 or 2500 instrument (Illumina Inc, San Diego, CA). Additional details describing contamination precautions, library preparation, and sequencing specific to each site are in the Supplementary Information.

### Statistical analyses of the full KEGG dataset

Reads were annotated through comparison to the KEGG database as described in the Supplementary Information. The resulting data are available at ScienceBase. Statistical analyses of the environmental dataset and the full KEGG dataset were carried out in R [30] and Primer-E version 6 [31]. The environmental dataset had 133 samples and 12 continuous variables (latitude, longitude, elevation, age, depth, total C, total N, C/N, organic C, pH, ice content, and EC) for a total of 1596 possible observations. Because our multivariate analysis methods exclude a sample if there is even a single missing observation, we used an expectation maximization (EM) algorithm to impute the missing values, as implemented in the Amelia R package [32]. We normalized all data to meet the assumptions of the EM algorithm (see Supplementary Information).

The imputed data were imported into Primer-E for PCA and PERMANCOVA analyses. A PCA of the environmental data was performed on the correlation matrix. PC1 primarily represented age, depth, TN, TC, OC, and pH (eigenvector coefficients: −0.31, −0.36, 0.45, 0.45, 0.44, −0.36) and explained 33.4% of the variation. PC2 primarily represented latitude, elevation, and C/N (eigenvector coefficients: 0.55, −0.43, −0.47) and explained 25.8% of the variation. The z-scores for PC1 and PC2 for each sample were output and used as covariates in subsequent PERMANCOVAs.

For PERMANCOVA analyses, we applied an abundance filter retaining only the genes observed more than 10 times in at least 10% of samples. Hypothesis testing was carried out using PERMANCOVA on KEGG gene relative abundance data, which were square root transformed and used to create a Bray-Curtis dissimilarity matrix. Two PERMANCOVA runs were performed testing the effects of PC1 and PC2 on gene distribution. An additional PERMANCOVA was also performed testing latitude as an a priori contrast fit to linear, quadric, or cubic orthogonal polynomial models. For all three PERMANCOVA tests, we used a 3-factor mixed, nested model with continent as a fixed factor and the random factors were nested (site within region and region within continent).

### Identification of highly variable functional genes

We identified KEGG genes showing the highest variability in abundance across sites using the DESeq2 package [33] in R [30]. Prior to this, we applied an abundance filter where genes observed more than 100 times in at least 10% of samples were retained. The *p* values were adjusted for multiple hypothesis testing using the Benjamini and Hochberg method. Sites with at least one replicate were compared to all other replicated sites, and the number of times each gene differed significantly (*p* < 0.01) between pairs of sites was counted. We defined highly variable genes as the top 5% differing most often between pairs of sites (ScienceBase Data). These genes were clustered using the ward.D2 method and visualized in the pheatmap package [34].

To determine if specific KEGG pathways, modules, or functional categories were significantly enriched in groups of highly variable genes identified by ward.D2 clustering, we used the hypergeometric distribution. We defined *N* as the total number of highly variable genes, *n* was the total number of genes in the ward.D2-identifed group, *K* was the total number of variable genes in a particular pathway/module/category, and *k* was the number of variable genes within the subgroup that were part of the particular pathway/module/category. The *p* value of finding *k* genes within a group was determined using the phyper function and corrected for multiple hypothesis testing using the Benjamini-Hochberg method in R.

### Relating taxonomic composition and potential function to biophysical parameters

Taxonomic classification was performed by extracting shotgun metagenome reads matching the V4 hypervariable region of the 16 S rRNA gene as described in the Supplementary Information. To understand the biophysical parameters that explained variation at the class level we performed constrained redundancy analysis (RDA) using the vegan [35] package in R [30]. For biophysical measurements, we used imputed and transformed data as described above. Taxonomic abundance data were Hellinger transformed. Linear dependencies between variables were assessed using the variation inflation factor (VIF). Multicollinearity was indicated by VIF values exceeding ten, so forward selection of explanatory variables was performed using the ordiR2step function. Significance of the remaining predictors was assessed using the anova.cca function and then corrected for multiple testing using the false discovery rate. Principal component analysis (PCA) on the transformed taxonomic abundance data was performed to compare constrained and unconstrained ordinations. We also regressed the relative abundance of the most abundant taxa against environmental variables (JMP 16, SAS Institute, Cary NC) including latitude, depth, pH, TC, OC, TN, electrical conductivity, and ice content.

We performed k-means cluster analysis on the highly variable KEGG genes and 16 S rRNA gene data (JMP 16, SAS Institute, Cary NC). For both KEGG and 16 S rRNA genes, we used a Johnson transformation and within cluster standard deviations to improve cluster assignments. We then quantified the optimal number of clusters using the elbow method, which minimized the sums of squares within clusters relative to the number of clusters examined. Clusters with four or fewer samples were removed from downstream comparative analysis using ANOVA. To examine whether different clusters were associated with different biophysical parameters, we utilized a one-way ANOVA with cluster as the main factor and biophysical parameters as the dependent variables. Prior to ANOVA we ensured that data were normally distributed by testing using a Shapiro-Wilk W Goodness of Fit test followed by normalization if necessary. Significant one-way ANOVA was followed by a Tukey-Kramer HSD test with an accepted *p* value of 0.05. Data are presented as means and one standard deviation unless otherwise stated.

## Results

### Soil physicochemical properties

In our panarctic investigation of 133 permafrost samples physicochemical characteristics ranged broadly. Our dataset represented samples from subarctic to high Arctic latitudes (Table 1). Permafrost age ranged from approximately one thousand years before present (kyr) to 880 kyr (ScienceBase). Most soils represent near-surface permafrost the upper several meters, but several locations contained deeper permafrost (>10 m) from tunnels or borehole samples. Pleistocene aged samples were deeper (11.9 ± 0.79 m vs 2.6 ± 0.9 m (standard error (s.e.)) than Holocene aged samples (*F*_1,131_ = 56.7, *p* < 0.001). Organic C ranged from approximately 1% to over 40% in frozen wetland soils. Holocene age permafrost soils had higher % OC compared to Pleistocene permafrost soils (9.2 ± 1.2 vs 2.6 ± 1.0 %) due to a large number of organic rich wetland soils contained in Holocene permafrost (*F*_1,128_ = 26.6, *p* < 0.0001). Similarly, %N was higher in Holocene aged permafrost (0.7 ± 0.07 vs 0.2 ± 0.06, *F*_1,126_ = 19.5, *p* < 0.0001 (s.e.)). C/N ratios varied widely, sometimes as low as 2 to as high as 23, with a mean of 13.2 ± 8.0 and was higher in Holocene than Pleistocene aged permafrost (16.0 ± 1.1 vs 10.8 ± 0.9, *F*_1,124_ = 14.5, *p* = 0.0002). Permafrost also ranged in soil pH. Soil pH differed by soil age (*F*_1,125_ = 37.5; *p* < 0.0001), with Pleistocene aged permafrost having a higher pH (7.3 ± 0.11) than Holocene aged permafrost (6.28 ± 0.13). Ice content ranged from 0.14 g/g to over 1.0, and did not differ by permafrost age (Table 1).

### Microbial taxonomic composition in permafrost across the Arctic

Analysis of 16 S rRNA gene sequences extracted from shotgun metagenomes demonstrated that permafrost bacterial and archaeal communities were highly variable in composition and diversity (Fig. 1B). Sequences affiliated with *Proteobacteria* and *Actinobacteria* dominated permafrost samples across the Arctic, averaging 41% and 20% of the total community, respectively. The distribution of other highly abundant taxa (*Firmicutes, Bacteroidetes*, and *Chloroflexi*) showed substantial variability across sites. For example, *Firmicutes* represented nearly 50% relative abundance in some interior Alaska samples (e.g. Fox Tunnel) but were vanishingly rare in others (Fig. 1B).

Shannon diversity was unimodally related to soil pH, where highest alpha diversity was observed in circumneutral pH soils and lower in acidic or alkaline soils (Fig. 1C). Several samples from the Vault Creek Tunnel, Wadepiper Lake, and Kolyma showed very low alpha diversity (Fig. 1C). Outlier samples from Vault Creek Tunnel tended to be deeper than much of the rest of the dataset, and the outlier from Kolyma was the oldest sample in our dataset (880 kyr).

Constrained redundancy analysis (RDA) demonstrated that environmental gradients explained variation in taxonomic structure at the class level. Permafrost microbial compositional variability was significantly explained by depth, latitude, elevation, pH, age, and yedoma classification (all *p* < 0.002, Fig. S1). We examined the distribution of the most abundant classes in relation to latitude, depth, and other factors. Regression statistics were corrected for multiple comparisons using the Benjamini and Hochberg method. With increasing latitude, *Actinobacteria* (*R*^2^ = 0.13, *p* < 0.008) and *Bacteroidia* (*R*^2^ = 0.05, *p* < 0.03) increased in relative abundance while *Alphaproteobacteria* declined (*R*^2^ = 0.08, *p* = 0.007). With increasing depth, *Gammaproteobacteria* declined (*R*^2^ = 0.06, *p* = 0.03). Permafrost age (Pleistocene vs Holocene) also affected the relative abundance of different taxa. *Bacilli*, *Alphaproteobacteria*, *Chlamydiia*, *Planctomycetia*, and *Chloroflexia* were more abundant in Pleistocene permafrost whereas *Gammaproteobacteria* and *Deltaproteobacteria* were more abundant in Holocene aged permafrost (Figure S2). At the level of phylum, *Acidobacteria* were also more abundant in Holocene permafrost. Soil pH, ice content, %OC, %N, and C/N did not consistently affect the relative abundance of many of these taxa across the panarctic.

### Variation in microbial functional gene in permafrost across the Arctic

To determine how functional genes were distributed across the Arctic, we annotated metagenome reads through comparison to the Kyoto Encyclopedia of Genes and Genomes (KEGG) database and evaluated the relationships between the relative abundances of KEGG genes, location, and biophysical factors. Permutational analysis of covariance (PERMANCOVA) tests indicated that interactions between biophysical factors and sampling location drove functional assemblages (Table S3). Covariates such as OC, N, and pH did not explain significant variation in the gene abundance data on their own but were significant when interacting with sample site. For the PERMANCOVA analysis that included the linear combination of %N, total C, % OC, age, depth, and pH as a single covariate (the first principal component (PC1) of the biophysical data), site nested within region explained 26.5% of the variation in gene relative abundance data (*Pseudo-F* = 6.36, *p* < 0.0001) and the interaction between PC1 and site within region explained 56.0% (*Pseudo-F* = 2.25, *p* = 0.0002). For PERMANCOVA analysis with the second principal component (PC2 representing a linear combination of latitude, elevation, and C/N) as a covariate, site within region explained 13.7% of the variation (*Pseudo-F* = 6.65, *p* < 0.0001) and the interaction between PC2 and site within region explained 77.3% (*Pseudo-F* = 2.48, *p* < 0.001). Tests to detect covariation between gene relative abundances and latitude (and whether patterns were linear, quadratic, or cubic) were not significant (*p* > 0.05).

### Clustering of samples based on highly variable functional genes and their relationship to environmental conditions

**Fig. 2:**
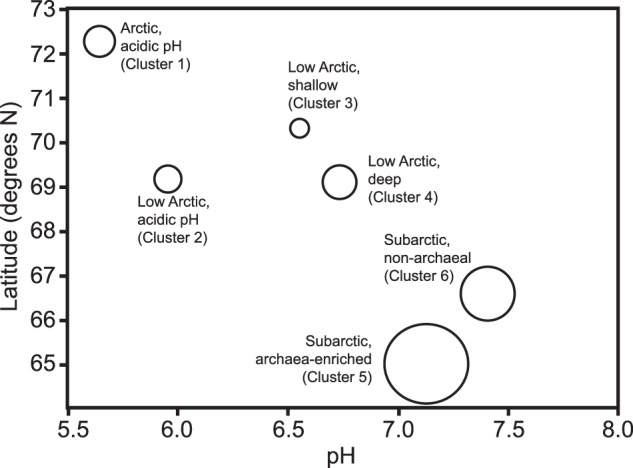
Six primary sample clusters vary by pH, latitude, and soil depth. Average pH for each cluster is shown on the x-axis and average latitude for each cluster is shown on the y-axis. Relative soil depth is indicated by circle size, in which larger size indicates greater depths.

We performed a selection procedure, identifying a subset of 327 genes from the original set that had the greatest variability among sites (top 5%, ScienceBase Data), which likely reflect the strongest selective pressures driving differences among microbial communities. We clustered samples based on the abundances of highly variable genes to determine whether clusters related to microbial functional potential exist across the Arctic and whether they are related to broad environmental gradients. This resulted in fifteen distinct clusters of samples. Six clusters had between six and 43 samples, which were retained for downstream analyses. Six clusters, each with an individual sample, and three clusters containing between 2 and 4 samples were removed from further comparisons. We used ANOVA to determine differences in biophysical properties among the remaining six clusters, which revealed that permafrost properties among clusters differed most significantly with respect to pH, latitude, and soil depth (significant one-way ANOVA, *p* < 0.0001, *R*^2^ > 0.26, Table S4, Fig. 2). Though we conservatively required clusters to have greater than six samples, this trend was similar even when all clusters were considered (Fig. S3). Permafrost age, %OC, and %N differed among clusters, but the variance explained was low (*p* < 0.05, *R*^2^ ≤ 0.19, Table S4). Clusters did not vary by continent, region, permafrost type (continuous vs discontinuous), C/N ratio, EC, or ice content. Nitrate + nitrite in all samples was relatively low, averaging 0.14 ± 0.26 mg kg^−1^ soil, and did not differ among sample clusters.

Clusters 1 through 6 were named and arranged according to environmental gradients (Table S4). For example, permafrost soils from low numbered clusters were more acidic, shallower, higher latitude, and richer in C and nitrogen (N) whereas permafrost soils from higher numbered clusters were from more alkaline, deeper, lower latitude, and lower C and N. Cluster 1 was above 70° N (Arctic) and Cluster 2 was from the low Arctic (between 67 and 70° N, Fig. 2). Clusters 3 and 4 were both from a more neutral pH and similar latitude (low Arctic) but differed by depth (Fig. 2). Clusters 5 and 6 from the subarctic (below 67° N), were the most alkaline of all clusters (pH > 7.0), and differed by soil depth (Fig. 2, Table S4). But clusters 5 and 6 also differed strongly in the relative abundance of archaea (15.3% versus 2.2%, respectively), which we used to label these two clusters (Fig. 2).

### Comparison of taxonomic composition and functional genes

We compared clusters of samples generated with 16 S rRNA gene abundance data with clusters of samples generated with functional gene profiles to determine whether taxonomic clusters corresponded to functional clusters. A contingency analysis indicated taxonomic clusters were not associated with functional gene-based clusters. The Agreement Statistic, based on the frequency of co-occurrence between taxonomic and functional gene clusters was very low (Kappa statistic = −0.01, *p* > 0.05) indicating that the observed agreement was less than by chance.

### Characterization of highly variable functional genes

**Fig. 3:**
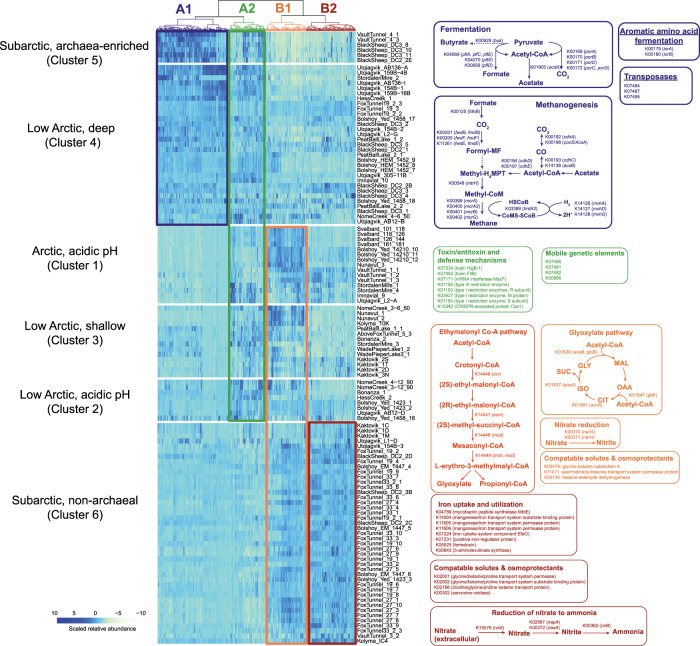
Heatmap and pathway diagrams of the most highly variable KEGG genes occurring across permafrost sites. Heatmap shows the scaled relative abundances of KEGG genes that were the most variable across sites, which were clustered using Ward’s minimum variance method. Genes clustered into two primary groups, A and B, which are shown with cool and warm colors, respectively. Subgroups within A are colored dark purple (A1) and green (A2). Subgroups within B are colored orange (B1) and red (B2). Samples (shown in rows) are those occurring in the six primary sample clusters and are organized by cluster for the purpose of visualization. They are labeled by cluster number and a description (including latitude, pH, depth, and archaeal abundance). Clusters 5 and 6 have a similar latitude and pH, and so are labeled by the relative abundance of archaeal genes (archaea-enriched versus non-archaeal) to differentiate between them. Colored boxes overlaid on the heatmap highlight the relationships between groups of genes and clusters of sites. Pathways from each gene group are shown next to the heatmap and are colored dark purple, green, orange, or red to indicate which group they belong to (A1, A2, B1, B2, respectively). Genes from the Ethylmalonyl Co-A pathway were found in Groups B1 and B2, which is indicated by dual coloring of the pathway with red and orange. KO numbers are followed by either a gene name or description in parentheses. The full list of highly variable genes with complete descriptions (shown in the order in which they occur in the heatmap) is available in ScienceBase. In pathway diagrams, dashed arrows indicate multiple steps that are not shown. For the glyoxylate pathway diagram, the abbreviations are as follows: glyoxylate (GLY), malate (MAL), oxaloacetate (OAA), citrate (CIT), isocitrate (ISO), and succinate (SUC).

We evaluated the distribution of highly variable genes among sample clusters, revealing that clusters had distinct metabolic and functional themes, which were associated with differences in energy metabolism, C assimilation, and substrate utilization (Table S5). Hierarchical clustering partitioned highly variable genes into two primary groups (A and B) with two sub-groups in each main group (A1, A2, B1, B2; Fig. 3, data available in ScienceBase). We refer to these as groups of functional genes rather than clusters to distinguish them from the clusters of samples described earlier. Group A1 (dark purple in Fig. 3), which contained genes that were highly abundant in Clusters 4 and 5, was significantly enriched in genes related to metabolism under reducing anaerobic conditions, specifically methanogenesis (16% of genes in A1, *p* < 0.0001) and fermentation (7.5% of genes in A1, *p* < 0.0001). Concomitant with the high abundance of methanogenesis genes (and therefore a high abundance of archaea), group A1 was significantly enriched in archaeal genes (e.g., archaea-specific elongation factors, polymerases, and translation initiation factors) (23% of A1 genes, *p* < 0.0001). The fermentation pathways in Group A1 produce end-products that fuel methanogenesis—formate, acetate, and CO_2_. These data indicate that the oxidative conversion of pyruvate to acetyl-CoA and CO_2_ is catalyzed by pyruvate ferredoxin oxidoreductase, formate is produced by pyruvate formate lyase, and acetate is produced by acetate-CoA ligase. Methanogenesis genes included both hydrogenotrophic and acetoclastic pathways (Fig. 3). The second set of genes in Group A (Group A2, indicated by green in Fig. 3) did not reveal a central metabolic theme. Instead, we observed a significant enrichment of genes related to toxin/antitoxin systems and host defense mechanisms (*p* = 0.0022), and transposases (*p* = 0.042). There is substantial uncertainty and debate over the function of toxin/antitoxin systems but they are often found on mobile genetic elements and may be involved in competition between cells [36].

Functional gene groups varied in the abundance of genes related to the metabolism of amino acids and other nitrogenous molecules, aromatic hydrocarbons, fatty acids, and carbohydrates (Table S5). The most notable trend in Group A was a greater number of genes related to carbohydrate degradation (*p* = 0.048) and, in Group A2, an enrichment of peptidases (*p* = 0.0022). Both of which were absent in Group B (Table S5).

Genes from Group A were highly abundant in the “subarctic archaea-enriched” and the “low Arctic deep” clusters of samples (Clusters 5 and 4, respectively) (Fig. 3). Clusters 4 and 5 contained samples from Blacksheep Pond, the oldest and deepest (~90 kya, 40 m) Vault Creek Tunnel samples, 19 kya section of the Fox Tunnel, Holocene permafrost from Bol’shoy Lyakhovsky Island, and Utqiaġvik polygons. Methanogens and methanogenic activity have been previously observed in 19 kya Fox Tunnel samples and Utqiaġvik polygons [9, 37, 38] and the Holocene Bol’shoy Island samples originate from ancient lake sediments, which may account for the high abundance of methanogenic pathways [39]. Shallow Blacksheep Pond samples are likely derived from a large ancient thermokarst lake system that existed at the site approximately 6 kya, which thawed through Pleistocene-aged yedoma. Prior to draining, methanogenic communities were likely established and subsequently incorporated into permafrost when it re-froze. Neither of the acidic sample clusters (Clusters 1 & 2) contained a high abundance of methanogenesis-related genes, which is consistent with the inhibitory effect low pH has on methanogenesis [40].

The genes in Group B are suggestive of more favorable redox conditions than genes in Group A. Nitrogen cycle genes were significantly enriched in Group B (*p* = 0.019) and are predicted to support anaerobic respiration using nitrate and nitrite as terminal electron acceptors through nitrate reduction to nitrite (*narG, narH*, and *napA*) and dissimilatory nitrate reduction to ammonia (DNRA) (*nirB*). We also observed evidence of the ability to conduct assimilatory nitrate reduction (*nasA*) and nitrate transport (*nrtA*) (Fig. 3).

Group B was also enriched in genes associated with anabolic pathways that use substrates entering central metabolism via acetyl-CoA (e.g., fatty acids, waxes, alkenes, ketogenic amino acids, methylated compounds, or C2 compounds such as acetate). This stands in contrast to molecules that enter through pyruvate or phosphoenolpyruvate, such as carbohydrates and most amino acids [41, 42]. Potential increased use of these substrates is represented by the significant enrichment of two anaplerotic pathways, the glyoxylate cycle and the ethylmalonyl-CoA pathway (*p* = 0.0022), which replenish citric acid cycle intermediates that have been drained for biosynthetic processes (Fig. 3) [43]. This observation is supported by the presence of fatty acid metabolism genes in Group B and their corresponding absence in Group A (Table S5).

Group B genes were associated with managing osmotic stress, such as the uptake and synthesis of osmoprotectants and compatible solutes (*p* = 0.0051) (Fig. 3). These compounds include glycine betaine, choline, and proline, and osmolytes that balance osmotic pressure, maintain turgor pressure, inhibit protein aggregation, and maintain membrane fluidity at low temperatures [44]. Group B also contained a transporter gene for spermidine/putrescine, which are small polyamines that protect cells against diverse stressors such as temperature, osmotic pressure, and reactive oxygen species [45].

Genes from Groups B were highly abundant in the “sub Arctic non-archaeal” cluster of sites (Cluster 6). These sites include most Fox Tunnel samples from the late Pleistocene 27 kya and 33 kya age categories, Eemian (125 kya) and some late Pleistocene (30 kya) permafrost samples from Bol’shoy Lyakhovsky Island in Russia, and some samples from Kaktovik, Alaska. Clusters 1, 2, and 3 (Arctic acidic, low Arctic acidic, and low Arctic shallow), were defined by a high abundance of genes from Groups A2 and B1. Metabolically, these clusters are more like Cluster 6 than Clusters 4 & 5 because of the low abundance of methanogenesis genes and high abundance of a subset of the nitrate reduction and anaplerosis genes. Notable samples from these clusters are from Svalbard, Kaktovic, Stordalen Mire, most late Pleistocene samples from Bol’shoy Lyakhovsky Island, and the youngest Vault Creek Tunnel samples (25 kya). Many samples from this metabolically similar group of clusters (i.e., 1, 2, 3, and 6) were previously found to contain a high abundance of acetate and/or other short chain fatty acids. Specifically, late Pleistocene (30 to 54 kya) Bol’shoy Lyakhovsky Island samples had high concentrations of acetate compared with Holocene samples [39]. Similarly, 27 kya and 33 kya Fox Tunnel permafrost samples had higher concentrations of acetate, butyrate, and isovalerate than 19 kya tunnel samples [4], which is consistent with our observations of an increased ability to assimilate these molecules.

## Discussion

Across the panarctic, permafrost microbial communities displayed significant site to site variability in both taxonomic and functional gene composition, much of which could be explained by latitudinal, soil chemical, and soil depth gradients. We observed latitudinal gradients in community composition and functional genes that are likely related to local climate, which we associate with a decline in mean annual air temperature with increasing latitude. Cold ground surface temperatures correspond to lower permafrost temperatures generally [46], with concomitant declines in substrate diffusion and microbial activity [47].

Soil pH had an impact on both the diversity of permafrost organisms and their functional genes. Soil pH strongly influences the structure and function of microbial communities in Arctic active layer soils and many other ecosystems worldwide [3, 48]. Significant variation in soil pH exists across the Arctic [48], and has been an important factor in structuring plant communities as well [49]. Soil pH may affect microbial diversity and their functional characteristics by modifying resource availability, energetics of reactions, and the presence of inhibitory substances [50]. We observed that in slightly acidic soils (<pH 6.25), functional genes associated with methanogenesis and fermentation were in relatively low abundance. For methanogens, this result could be due to the direct inhibitory effect of pH, particularly on acetoclastic methanogens [51].

Soil depth was a major influence on the taxonomic composition and functional genes of permafrost microbial communities as well. Increasing soil depth likely adds stress to microbial communities due to lower resource availability [52]. Deeper permafrost soils (10 s of meters) had lower C and N concentrations compared to shallow permafrost. But other factors such as lower permafrost temperatures, lower rates of diffusion, and older permafrost age may also be an important contributor to this depth dependent pattern [53, 54].

Several other factors, particularly permafrost age, soil OC, and soil N, had a significant influence on permafrost taxonomic composition and functional genes, but the amount of variability explained was lower than we expected. We had hypothesized that these factors, including the soil C/N ratio, would have more of an influence over taxonomic or functional clusters, as observed in another study [55] and given the importance of C/N ratios in governing rates of C fluxes from thawed soils [56]. This incongruity may come from the fact that permafrost microbial communities only access a small portion of the permafrost soil C and N pool because they survive and grow in narrow water films [57]. Thus, bulk chemical measurements may not represent the fraction of permafrost C and N being accessed by the community prior to thaw. Permafrost age is particularly complex because it covaries with both soil depth and soil pH. Our Pleistocene samples were deeper and more alkaline than Holocene aged permafrost. This could be in part because Pleistocene permafrost is more often associated with loess hills while Holocene permafrost is more often located near lowlands. Regardless, once Pleistocene permafrost thaws, soil pH could become more acidic as carbonates are removed [58]. Thus, Pleistocene permafrost that thawed and reformed in the Holocene could be more acidic on average, indirectly producing the pH-dependent functional gene clusters observed in this study.

Though we found that environmental parameters had a significant effect on both taxonomic and functional genes, the strengths of these effects differed among data types. The strongest patterns were observed in the subset of highly variable functional genes. There are likely several explanations why highly variable genes show the strongest relationships with biophysical attributes of soils. First, they represent genes experiencing the strongest selective pressures. Second, by excluding genes that did not vary (e.g., housekeeping genes) we likely enhanced detectable signals of variation across the landscape. Furthermore, differences in association with environmental parameters between taxonomic and functional genes could be partially explained by functional redundancy [59]. In this case, functional genes may be strongly associated with environmental parameters, with little change in taxonomic composition. We also acknowledge the limitations of short reads in performing taxonomic identifications. We conservatively chose to limit analyses to class, which is a somewhat coarse level of resolution. It is possible that genus or “species” level classifications would yield a more complete view of the relationships between environment, genes, and taxonomy.

### Conceptual model of permafrost microbial community assembly

**Fig. 4:**
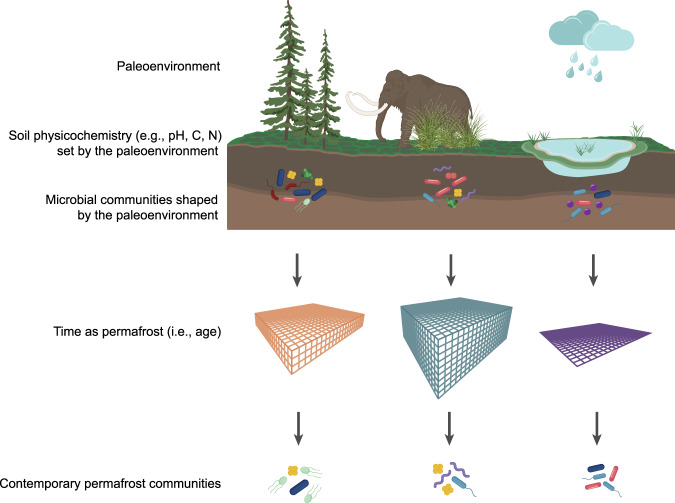
Proposed model of permafrost microbial community assembly. Paleoenvironmental factors, such as plant community structure, climate, mechanism of permafrost formation, and water regimes shape soil microbial communities. Paleoenvironment also establishes soil physicochemical conditions within permafrost that, in combination with static subzero conditions, act through time to shape modern communities. Filter size reflects time since permafrost formation and filter color indicates differing environmental conditions. Immigration into permafrost is limited because subzero temperatures restrict water flow, so contemporary microorganisms are largely a subset of past communities that have undergone environmental filtering and diversification. Figure created in Adobe Illustrator and Biorender.

Based on the patterns of taxonomic composition and functional gene abundances, we propose a conceptual model of permafrost microbial community assembly (Fig. 4). Just as environmental variation creates differences in microbial community structure and functional gene distribution in non-permafrost soils [1–3, 60], the paleoenvironment prior to and during permafrost formation shaped communities in the soils that eventually become permafrost [4, 61, 62]. The permafrost formation process itself, which is driven by the coupled effects of paleoclimate (e.g., warm and wet, cold and dry) and paleoecosystem (e.g., forest, grassland, lake sediment), also affects OM characteristics [63, 64] and likely the corresponding microbial communities [10]. For example, syngenetic permafrost generally experiences fewer freeze-thaw cycles than epigenetic permafrost, which may lead to a more labile C pool because of reduced exposure to microbial processing prior to formation [4]. On the other hand, epigenetic permafrost, which can take millennia to form [5], may contain a more decomposed C pool [65].

Once entrained, frozen conditions are an environmental filter as a barrier to the import of resources, water, and microbial immigrants. But this filter also varies because microorganisms may remain active, especially in permafrost where temperatures approach 0 °C [66], transforming the community and the resources surrounding it [4, 9, 67, 68]. The longer microorganisms are entrained in permafrost, the further these compositional and functional transformations can occur. Therefore, contemporary permafrost communities are a subset of ancient communities that have passed through selective filters or survived through stochastic processes (e.g., drift) and may have diversified since entrainment depending on permafrost age, resource availability, and temperature (which affect cell division rates and growth).

### Functional genes differentiating permafrost microbial communities

The most highly variable genes across all permafrost samples were overwhelmingly related to energy acquisition and substrate availability suggesting that soil redox conditions, OM pools, and thermodynamic constraints on microbial metabolism are some of the strongest selective pressures affecting permafrost microbial communities. Co-occurrence of fermentation and methanogenesis genes underline that fermenters degrade labile organic C and produce the acetate, formate, and CO_2_ that supply methanogens. On the other hand, genes and pathways abundant in the non-methanogenic clusters indicate more favorable redox conditions. DNRA and nitrate to nitrite reduction genes indicate nitrate may serve as a thermodynamically favorable terminal electron acceptor to fuel microbial oxidation of OM through anaerobic respiration. While nitrate reduction functional genes show significant site to site variability, other abundant N cycle genes were more uniform in their distribution. For example, the relative abundance of *norB* (responsible for nitric oxide reduction to nitrous oxide (N_2_O)) was similar to the highly variable *narG* (nitrate reduction to nitrite) but *norB* relative abundance was comparatively stable across sites (see Supplementary Information). N_2_O is a potent greenhouse gas and thawing permafrost may be a significant source in the coming decades and centuries [69]. Our data suggest that microorganisms with the potential to produce N_2_O are widely distributed in permafrost. This, coupled with the observation that *nosZ* (catalyzes the final step in denitrification–N_2_O to N_2_) is rare in our samples indicates that permafrost microorganisms are poised to produce N_2_O and may currently be doing so.

Differences in functional gene groups also suggest pathways of OM transformations in permafrost may vary across sites. The high relative abundance of genes in the ethylmalonyl-CoA pathway, the glyoxylate shunt, amino acid and fatty acid metabolism in non-methanogenic permafrost could indicate microorganisms are unable to access carbohydrate-rich OM and are required to use less energetically favorable substrates. The high abundance of the glyoxylate shunt may also be an indicator of quiescence, where cells are viable and metabolically active but persist in a non-replicating state [70]. Another recent study from the coastline of the Eastern Siberian Sea similarly found that metabolic strategies were key to adaptation to cold anoxic permafrost environments and even identified many of the same genes (e.g., nitrate reduction, peptidases, and fermentation) that differentiated permafrost communities from distinctive depths and ages [11].

Because functional gene profiles indicate potential function, we do not know how these relate to actual function in intact permafrost and post thaw, particularly because microbial communities can quickly change after transition from a frozen to thawed state [16, 71]. Furthermore, we did not differentiate between active, dead, or dormant microorganisms so we are limited in our view of who is active or who may become active. However, we did not observe substantial differences between live and dead populations within intact permafrost in a previous study [72]. Cluster analysis, although useful to understand similarities among samples, does not perfectly aggregate samples within sites. We observed instances where samples from the same site were associated with different clusters, which likely indicates that sometimes local scale variability outweighs regional differences. Furthermore, the biophysical descriptors associated with each cluster are averages and individual samples may be from differing environments. Lastly, much of the available data on permafrost microbial communities, including data from this study, are biased towards Alaska and even particular sites within Alaska. Future efforts should increase representation from Canada, Europe, and Asia and systematically target geographically, geologically, and ecologically distinct permafrost environments using well-replicated sampling designs.

Though we identified environmental factors that contribute to shaping permafrost microbial community functional potential, there are still gaps in our understanding that limit our ability to make predictions about the distribution of functional gene groups based on permafrost characteristics. Incorporating information about paleoenvironment and site history may be important keys to predictive understanding of the distribution of microbial communities and their functions in permafrost. For example, understanding paleovegetation may fill some of our knowledge gaps. Permafrost microbial communities should show adaptations related to characteristics of the plant community present at the time of formation. Prior studies from the Fox Permafrost Tunnel metagenomes provide support for this argument. Paleovegetation (inferred from metagenomic sequences derived from plant detrital DNA) was correlated with historical climate, dissolved organic C characteristics, and the abundance of genes encoding carbohydrate active enzymes [4, 9]. In the future, expanding studies to diversify permafrost environmental locations coupled with functional and biogeochemical measurements and site history investigations promises to enable predictive understanding of global patterns and will enable us to address increasingly complex questions about the principles governing organization of microbial life in permafrost and the effects of these communities on crucial biogeochemical cycles, both currently and in response to climate change.

In soils, approximately 40% of DNA is from cells that are no longer intact [73]. In permafrost, the proportion of recovered DNA from dead cells may be even greater because frozen conditions act as a preservative [74]. In samples from the Fox Permafrost Tunnel, depletion of exogenous DNA decreased yields by 24–62% depending on sample age [72]. The extent to which DNA from dead cells alters conclusions in soil DNA-based studies is an open question. For this study, we suggest that the overarching conclusions would likely not change, though some of the smaller details might. Previous work in permafrost suggests that removal of relic DNA does not substantially alter microbial community structure [72]. This result is expected when the death rate and degradation of detrital DNA is similar across taxa. Similar observations have been made in deep marine sediments [75] which, like permafrost, host communities that must survive in a cold environment with limited resources.

### Concluding remarks

Permafrost is poised to be one of the largest biological feedbacks to future climate change [76]. However, our limited understanding of permafrost and its microbial inhabitants may hinder our understanding of system feedbacks and representation in climate models, jeopardizing efforts to predict the consequences of permafrost thaw in a changing climate. The fact that permafrost soils are not a monolith is a hurdle to our understanding: they are incredibly diverse in their paleoecology, geology, soil chemistry, and soil microbiology. Permafrost soil microorganisms have the unique feature of containing information about both the paleoenvironment and the modern environment, which can be interpreted through their genetic composition. The microorganisms in thawed permafrost soils may be derived from the permafrost community, thereby having an outsized effect on soil function. Despite enormous diversity in permafrost environments and variation in community structure and functional genes, we identified large scale patterns. The entrainment of microorganisms in permafrost during its formation, coupled with frozen static conditions and barriers to immigration, likely makes paleoenvironment a crucial yet overlooked factor in the structure of contemporary communities [65]. These microorganisms are poised to act as “first responders” during thaw, controlling turnover of the most labile and accessible substrates [67], and greenhouse gas fluxes. In this way, past legacies will uniquely shape future ecosystem responses.

## Supplementary information

Supplemental Material

## Data Availability

Datasets including biophysical data, taxonomic data, and KEGG data are available on ScienceBase at 10.5066/P9ZDRJ3K. Sequence data are available at the National Center for Biotechnology Information (NCBI) under BioProject accession number PRJNA830449.
