# Supplementary material for: Permafrost microbial communities and functional genes are structured by latitudinal and soil geochemical gradients
Source: ISME J. 2023 May 22;17(8):1224–35. doi: 10.1038/s41396-023-01429-6 (PMC10356821; doi:10.1038/s41396-023-01429-6)
Supplement: Supplementary file 1 — Supplemental Material [file 41396_2023_1429_MOESM1_ESM.docx]

**Supplementary Information for**

**Permafrost microbial communities and functional traits are structured by latitudinal and soil geochemical gradients**.

Mark P. Waldrop^*^, Christopher L. Chabot, Susanne Liebner, Stine Holm, Michael W. Snyder, Megan Dillon, Steven R. Dudgeon, Thomas A. Douglas, Mary-Catherine Leewis, Katey M. Walter Anthony, Jack W. McFarland, Christopher H. Conaway, Christopher D. Arp, Allen C. Bondurant, Neslihan Taş, Rachel Mackelprang^*^

*Corresponding Authors: Rachel Mackelprang and Mark Waldrop

Emails: [rachel.mackelprang@gmail.com](mailto:rachel.mackelprang@gmail.com) and [mwaldrop@usgs.gov](mailto:mwaldrop@usgs.gov)

**Extended Methods**

***Site locations***

*Fox Permafrost Tunnel*

We collected permafrost cores in October 2016 from the Cold Regions Research and Engineering Laboratory (CRREL) permafrost tunnel (also called the Fox Permafrost Tunnel) near Fairbanks, Alaska (64.951 °N, 147.621 °W). The tunnel penetrates a hillside, providing access to Pleistocene permafrost [1]. It is located near the valley floor of Goldstream Creek in a region of discontinuous permafrost.  Permafrost here is ice-rich silt of aeolian origin (loess), which was formed syngenetically through sediment deposition causing permafrost to build upwards over geologic time. We collected cores from three locations along the tunnel wall corresponding to age categories that had previously been determined using radiocarbon dating [2].  Before coring, loose silt resulting from sublimation was scraped away to expose fresh permafrost material. Using a sterilized round 10 cm diameter hole saw attached to a power drill, replicate cores were collected approximately 10 cm apart. Cores were placed immediately in coolers with dry ice and remained frozen during transport.  To sample the 5 kya permafrost, we used the SIPRE corer to collect permafrost from above the tunnel at a depth of 67-82 cm from the surface. The sample collection description here applies to cores 5-10 from each age category. Cores 1-4 were previously described [2].

*Vault Creek Permafrost Tunnel*

We collected cores in March 2017 from the privately owned goldmining Vault Creek Permafrost Tunnel (65.029 °N, 147.698 °W), situated 20 km north of Fairbanks and approximately 10 km NNW of the CRREL Permafrost Tunnel. Cores were collected using the same techniques as with the CRREL Permafrost Tunnel.  The tunnel was sampled at four discreet locations along its length corresponding to approximate ages: 25 ka (31 m from tunnel portal), 42 ka (50 m), 50 ka (153.5 m), and 268 m from entrance (approximately > 90 ka) as determined in [3, 4]. The 268 m sample was from new excavation at the bottom of the tunnel and beyond what was available for sampling in [4]. Similar to the CRREL tunnel, permafrost here is syngenetically-formed ice-cemented loess.

*Blacksheep Pond*

We collected permafrost cores in March 2017 from the Goldstream Valley, approximately 11 km north of Fairbanks, AK, using a truck-mounted drill that operated without drilling fluid. Samples ranged 5 to 45 m depth. Samples were collected from two sites near Blacksheep Road, called Deep Core 2 (BSP-DC2; 64.8883 °N, 147.9204 °W) and Deep Core 3 (BSP-DC3; 64.8903 °N, 147.91634 °W). Deep core 2 was obtained from Blacksheep Pond, a 0.83 m deep small thermokarst pond that formed after 1949 along the margin of a larger drained lake basin. Beneath the pond, a ~5 m thick talik is underlain by permafrost sediment, which extends 43 m below the ground surface. Permafrost sediments consist of organic rich silt with abundant wood and aquatic plant macrofossils, the remnants of the first-generation lake’s refrozen sediments. Radiocarbon-dated terrestrial plant macrofossils suggest the original lake existed around 6,000 years ago. Subsequent drainage followed by refreezing of the talik would have resulted in the ice-poor, organic rich permafrost sediments observed in the borehole. Deep Core 3 was obtained 300 m north of Blacksheep Pond, 65 m south of Goldstream Creek. Deep Core 3 consisted of 40 m of ice-poor frozen silt and sand characteristic of the region’s Pleistocene-aged yedoma. Like Deep Core 2, Deep Core 3 lacked massive ice wedges. This together with its location in the flat basin, suggests it too may have been part of the ancient drained lake.

*Peatball Lake and Wadepiper Lake*

We collected permafrost from the Outer Coastal Plain of Alaska in the Teshekpuk Lake Wetlands using a SIPRE corer in April 2016.  We cored permafrost from upland sites (PBC1) adjacent to Peatball Lake (70.710 °N, 153.938 °W), and the center of low centered polygons (PBC2). At a second lake, Wadepiper lake (70.513 °N, 153.869 °W) we collected samples from low centered polygons (WPC1) on the eastern side of the lake (possibly a remnant drained lake basin), as well as samples from sub-lake permafrost (WPC3) from beneath the lake.

*Barter Island (Kaktovik)*

In September 2014, cores were collected from a cliff face on the shore of Barter Island, AK (70.1329 °N, 143.6881 °W) near the village of Kaktovik.  Permafrost within the coastal bluffs consists of layers of sandy silt below the surface peat layer [5]. We removed small permafrost cores using a sterilized sharpened core barrel attached to a handheld battery-operated power drill from a clean exposed permafrost surface on a cliff face after removing debris from the cliff face that had slumped over. Samples were collected from 0.75 m to 2.3 m in depth from the land surface.

*Utqiaġvik*

Permafrost samples were collected from the Barrow Environmental Observatory (BEO) which is located approximately 6 km east of Utqiaġvik, AK (71.3° N, 156.5° W) through the Next-Generation Ecosystem Experiment Arctic (NGEE Arctic) project [6]. This research site is characterized by thaw lakes, drained thaw lake basins, and ice-wedge polygons that cover more than half of the land surface [7]. The frozen permafrost cores were collected from high, transitional, and low polygon futures with a SIPRE soil corer during the late winter months (April–May) of 2013-2016 via multiple-trips. After retrieval, the cores were packed with dry ice in coolers and transported to the Lawrence Berkeley National Laboratory where they were kept in −25°C freezers for storage until analysis [6].

*Bol’shoy Lyakhovsky Island*

Permafrost cores from Bol’shoy Lyakhovsky Island, located between the East Siberian and Laptev seas in Russia, were collected in April 2014 using a KMB-3-15M rotary drill rig. DNA was obtained from subsamples of the cores described in detail in [8]. Briefly, samples were selected to represent a chronosequence from the Holocene to the Eemian interglacial period (~115-130 ka). Eemian samples derive from thermokarst lake sediments deposited when the climate was warm and wet with small ice sheets and high sea levels. Pleistocene samples represent carbon-rich fine-grained Yedoma permafrost. Holocene samples originate from alas lake (shallow depression primarily found in Yakutia, Russia formed from melting and refreezing of permafrost) deposits, which combined with climate, make their origins more like the Eemian rather than Pleistocene samples.

*Publicly available datasets*

We downloaded the remaining datasets using public repositories (Table S1). Atqasuk samples are from an area of continuous permafrost in northern Alaska, 90 km south of the city of Utqiaġvik and 1.5 km west of the Meade River [9]. Bonanza samples are from the Bonanza Creek Long-Term Ecological Research Program near Fairbanks, AK [10]. The Eureka samples were collected from the Canadian high Arctic, near the settlement of Eureka on Ellesmere Island [11]. Hess Creek samples represent lowland organic carbon (OC) rich permafrost from interior AK, south of the Yukon River near the Dawson Highway [12]. Imnavait samples were collected from the Imnavait Creek watershed on Alaska’s North Slope [13], overlain by moist acidic tussock tundra. Kolyma samples were collected from the Kolyma-Indigirka Lowland in northeastern Siberia [14]. Nunavut samples were obtained from the McGill Arctic Research Station on Axel Heiberg Island in Canada and were characterized as low OC mineral soils [15]. Permafrost samples from the Stordalen Mire study area in Northern Sweden represent discontinuous permafrost overlain by well-drained palsas [16, 17]. Nome Creek samples are from an upland boreal forest 100 km northeast of Fairbanks, AK in the southeastern portion of the White Mountains National Recreation Area [18]. Svalbard permafrost samples were obtained from an ice-wedge polygon site in the Adventdalen Valley in Svalbard, Norway. Four permafrost layers were collected at the following depths: 101, 118, 126, and 161 cm [19].

**Chemical analyses**

Samples analyzed by the USGS were measured as follows. Total soil C and OC and nitrogen (N) were measured on dried subsamples that were powdered in a modified roller mill and analyzed using combustion analysis on a LECO 2000 CNS autoanalyzer (LECO, St. Joseph, Michigan, USA).   Ice content (g /g dry soil) was determined following oven-drying at 70°C to a constant mass. pH was determined from a 1:2 (mass:volume) slurry of soil and deionized water equilibrated at room temperature for 30 min (AB15 pH meter, Fisher Scientific). Soil electrical conductivity (EC) was measured on the same 1:2 slurry following an additional equilibration time of 2 h (CDM80, Radiometer Analytical, Lyon, France). For previously published samples, these data were extracted from the literature (see citations in Table S1).

For Utqiaġvik samples, soil pH and electrical conductivity (EC) were measured on slurries using standard Sper Scientific pH and EC meters. Soil moisture was measured as the change in mass after 48 h drying at 105°C. Total OC and N were determined via dry combustion with direct measurement by MACRO cube elemental analyzer (Elementar, Langenselbold, Hesse, Germany). The estimated age range of permafrost at this location determined by radiocarbon is between 8,200 and 14,000 years [20].

**Core sub-sectioning and DNA extraction**

For the new Fox Tunnel samples (cores 5-10), Vault Creek Tunnel, Blacksheep Pond, Kaktovik, Peatball Lake, and Wade Pieper Lake, sampling equipment was sterilized using 10% bleach followed by 70% ethanol spray prior to sub-sectioning and handling. Cores were handled only on sterile aluminum foil and all tools (e.g., forceps, knives, chisels) were sterilized prior to use and between each sample. To mitigate sample contamination by modern DNA or due to differences in sample collection techniques, the outer 0.5 cm of each core was scraped off using sterile knives and chisels prior to DNA extraction [2]. DNA was extracted from a 0.5 g subset of the cleaned core using the FastDNA SPIN Kit for soil (MP Biomedicals, Santa Ana, CA, USA) according to the manufacturer’s protocol, with the addition of a clean-up and purification step using the DNeasy PowerClean Pro Cleanup Kit (Qiagen, California, USA). DNA quantity was assessed using the PicoGreen dsDNA Assay Kit (Thermo Fisher Scientific Technologies, Wilmington, DE).

For Utqiaġvik samples, each core was first sliced into subsections of 10 cm in length with a rotary saw in a cold room (−18 °C). Then these subsections were further cut to remove potential surface contaminants via removing outermost 2 cm using sterile blades. To extract DNA, we used the MoBio PowerSoil (current name: DNeasy PowerSoil Kit, Qiagen, Germantown, MD, USA) of 0.5-2 gr with minor modifications as follows. Prior to bead-beating, the samples were incubated in bead-solution at 65 °C for 5 min. Samples were disrupted by bead beating with the FastPrep ® Instrument (QBIOgene, Carlsbad, CA) at a setting of 5.5 for 45 s and the DNA was further purified according to the kit protocol. DNA amounts were quantified using the Qubit dsDNA HS assay (Invitrogen, Carlsbad, CA, USA).

For the samples from Bol’shoy Lyakhovsky Island, the outer portion of the cores were removed and discarded so that only material without contact with the drilling rig was used. DNA was extracted using FastDNA SPIN Kit for soil (MP Biomedicals, Santa Ana, CA, USA) according to the manufacturer’s protocol. DNA was quantified using the Qubit dsDNA HS assay (Invitrogen, Carlsbad, CA, USA).

**Library preparation and sequencing**

For the Fox Tunnel, Vault Creek Tunnel, Blacksheep Pond, Kaktovik, Peatball Lake, Wadepiper Lake, and Bol’shoy Lyakhovsky Island samples, sequencing libraries were prepared using an emulsion PCR (emPCR) protocol as described previously (Mackelprang et al. 2017, 2011). Paired-end sequencing (2x100 bp) was performed on an Illumina HiSeq 4000 instrument at the University of Chicago Genomics Facility.

For Utqiaġvik, total metagenomic DNA was sheared using the Covaris S-Series instrument (140 PIP, 10.0 duty factor, 200 cycles/burst, and 65 s) (Covaris, Woburn, MA, USA). Sequencing libraries were prepared using Illumina TruSeq DNA sample preparation kit v2, following the low-throughput protocol (Illumina Inc, San Diego, CA). Libraries were sequenced at the Department of Energy Joint Genome Institute on the Illumina HiSeq 2500 instrument to generate paired 150 bp reads.

For all metagenomes, we performed adapter trimming and quality filtering using BBDuk, a package within BBTools, using recommended parameters (BBMap – Bushnell B. – sourceforge.net/projects/bbmap/). For metagenomes with paired end sequencing, FLASH was used to combine paired reads with the max overlap flag set to 85 (Magoč and Salzberg 2011). A database of potential contaminants was generated from sequencing negative controls and were removed using MIRABAIT within MIRA v4.0.2 (http://sourceforge.net/projects/mira-assembler/).

**Taxonomic classification and functional annotation**

We performed taxonomic classification by extracting 16S rRNA gene sequences from shotgun metagenome data. We compared sequence reads to the V4 hypervariable region of the 16S rRNA gene, which was selected to improve comparisons between these data and amplification-based 16S rRNA gene surveys that predominantly target this region. V4 sequences were extracted from the SILVA SSU NR 99 132 reference database (Quast et al. 2013) and matching reads were identified using the UBLAST function from USEARCH-64 v7.0 with an e-value cutoff and acceleration set to 1e-6 and 0.4, respectively [21]. Taxonomic frequency estimations were performed in MEGAN 6.18.0 at default parameters using the SSURef_Nr99_132_tax_silva_to_NCBI_synonyms.map file [22]. For each sample, only bacterial and archaeal taxa with a mean number of assignments greater than two were used. We conservatively limited classifications to the levels of phylum and class (Science Base) because our reads are shorter than those typically generated through amplicon sequencing. Because of the short length, we were unable to achieve class-level resolution for some of the reads. In these instances, we extracted phylum-level classifications. Alpha diversity at the class level was calculated using the Shannon diversity index within the R [23] phyloseq package [24]. The class-level calculation likely underestimated diversity but was necessary (in contrast to using unique sequences) because the short reads result in non-overlapping fragments.

To annotate reads, we compared sequences to the KEGG database (Release 77.1) [25], using DIAMOND (v0.0.19) [26] with an e-value threshold of 1e-6, as described previously [2]. Reads were assigned the KEGG Orthology (KO) number of the top hit. From the top hits, we generated gene count matrices that include the number of times each KO was observed in every sample (ScienceBase).

**Statistical analyses**

Missing environmental variables were imputed using the Amelia package [27] in R [23] which operates by maximizing the likelihood of the estimate for each missing observation based on patterns in the variables and correlation with other variables. This method is generally acceptable when fewer than 5% of observations are missing and when there are more than seven data points per parameter [28]. Our data were within threshold values (<5% of observations were missing, and on average 126 data points per parameter were observed), indicating that this was an appropriate application of the EM algorithm.

The relative abundances of nitrogen cycle genes (*narG, nosZ, and norB)* were compared by normalizing the read counts by the average length of each gene in the KEGG database. This enabled direct comparisons despite differences in gene lengths.

**Supplemental Figures**


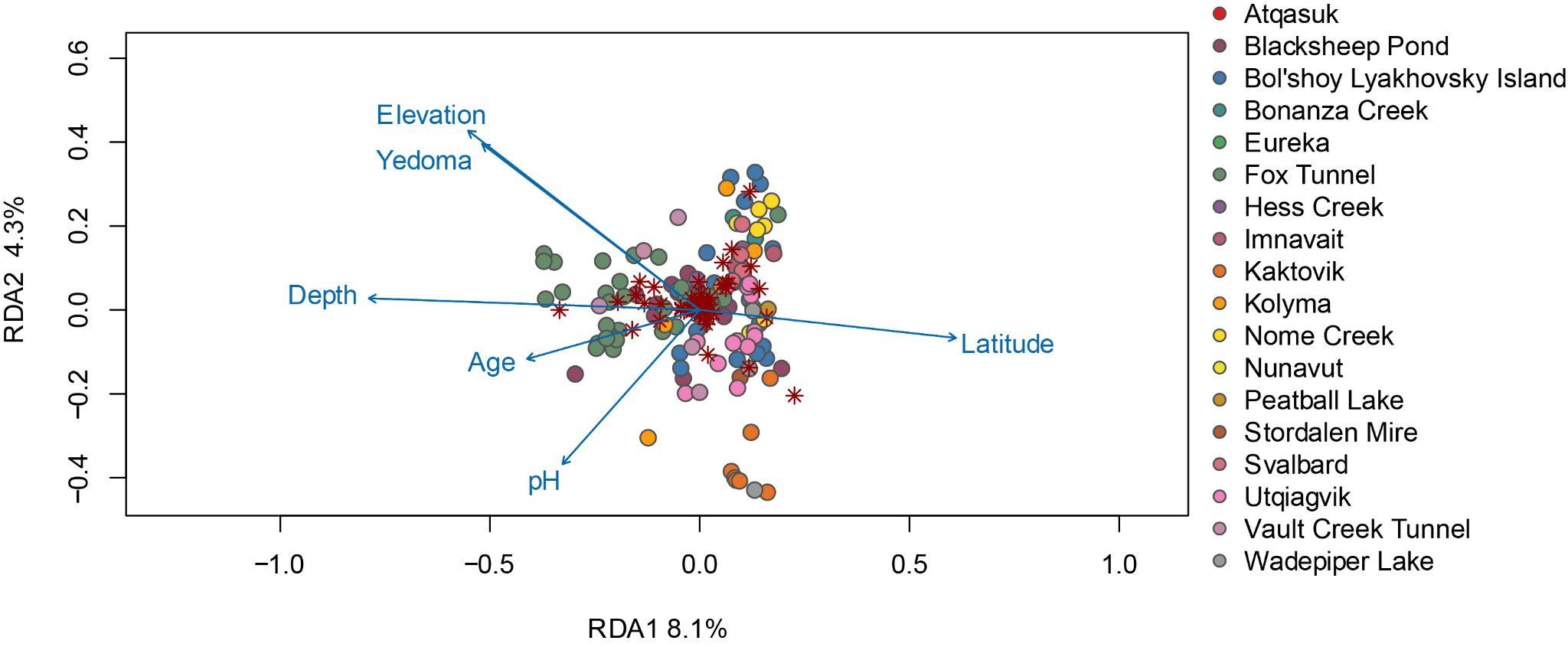


**Figure S1.** Redundancy analysis (RDA) triplot (scaling 3) based on 16S rRNA gene abundance data the class level and constrained by the highest-loading environmental variables. Circles represent sites colored to show location and red stars show bacterial and archaeal classes.


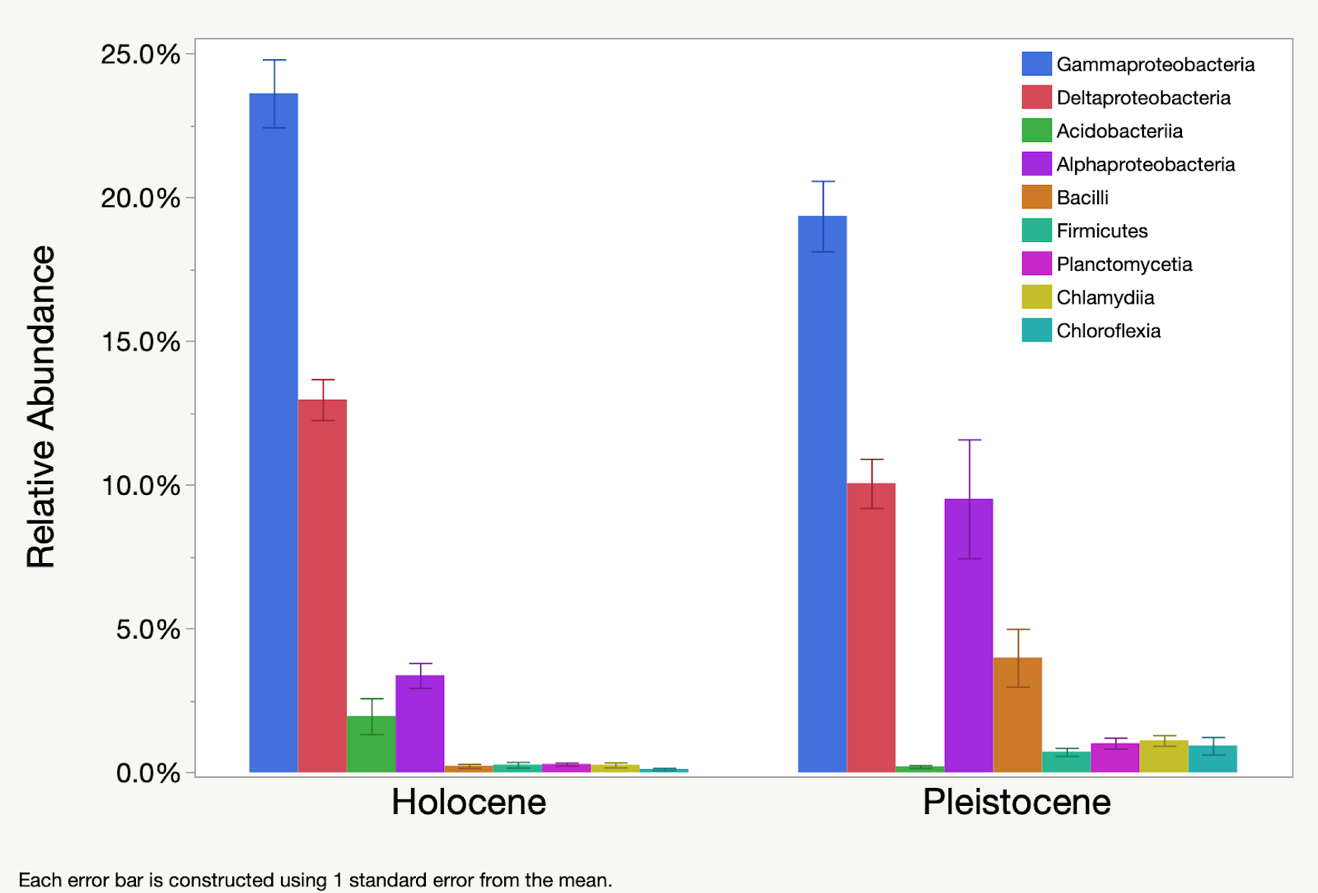


**Figure S2.** Relative abundance of major groups of organisms in different aged permafrost soils. Values are means ± 1SE.


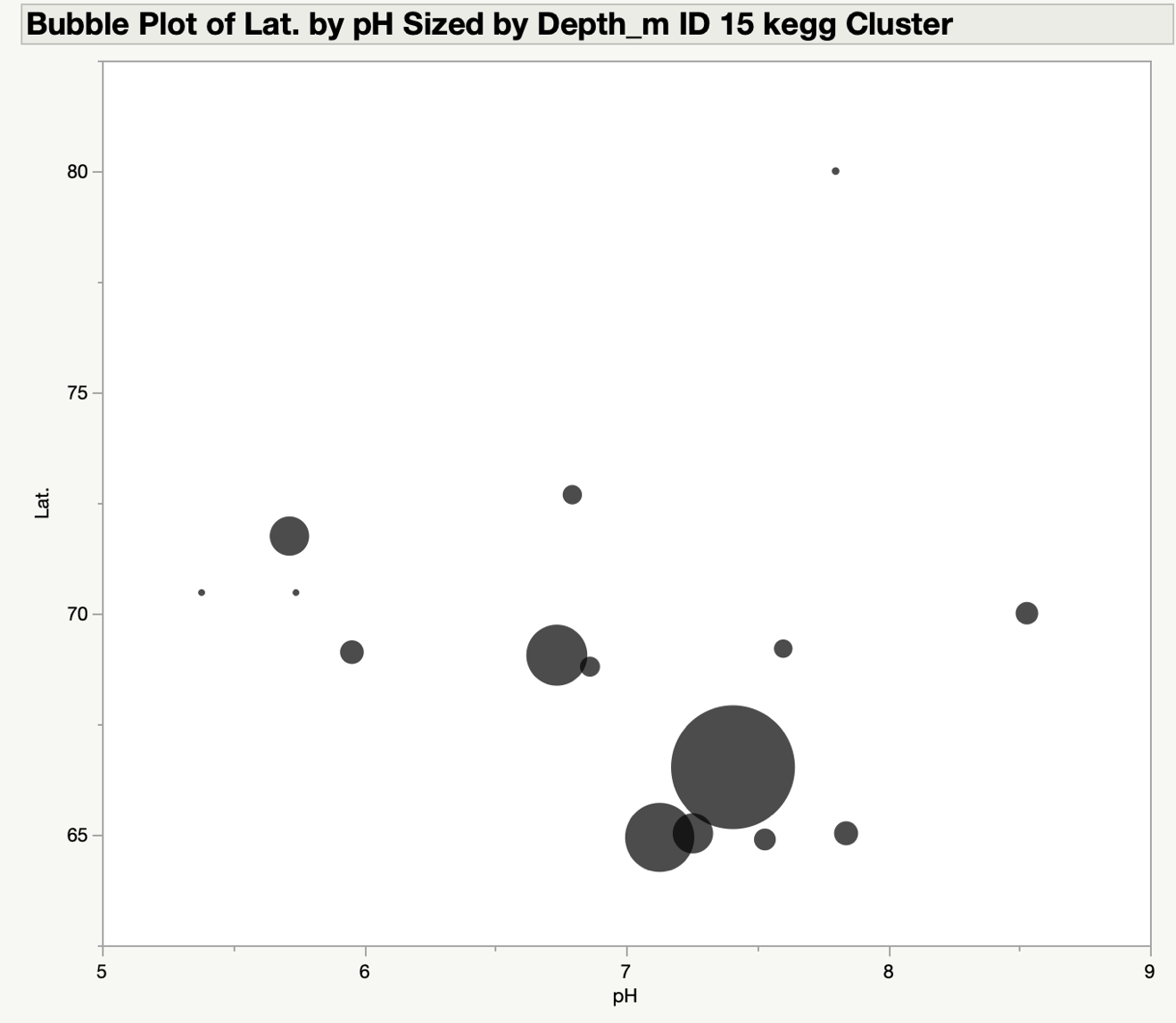


**Figure S3**. Bubble plot sample clusters based on highly variable KEGG genes. Shown is the differentiation of clusters by average latitude, soil pH, and depth. Average depth is indicated by bubble size, with larger bubble indicating deeper samples. Many of these clusters had n < 4, and thus were removed from primary analyses (see manuscript Figure 2).

**Supplemental Tables**

**Table S1. Accession numbers for previously published permafrost metagenomes.** Accession number starting with S are from the National Center for Biotechnology Information (NCBI) database. Accession number starting with Gb are from the Genomes Online Database (GOLD).

| **SampleID** | **Accession** |
| --- | --- |
| Atqasuk_AQ5D | SRX263030 |
| Atqasuk_AQ6D | SRX263031 |
| Bonanza_1 | Gb0051913 |
| Bonanza_2 | Gb0051952 |
| Eureka | SRR034818 |
| FoxTunnel_19_1 | SRX2368866 |
| FoxTunnel_19_2 | SRX2372151 |
| FoxTunnel_19_3 | SRX2372158 |
| FoxTunnel_19_4 | SRX2372159 |
| FoxTunnel_27_1 | SRX2372160 |
| FoxTunnel_27_3 | SRX2372161 |
| FoxTunnel_27_4 | SRX2372162 |
| FoxTunnel_27_5 | SRX2372163 |
| FoxTunnel_33_1 | SRX2372169 |
| FoxTunnel_33_2 | SRX2372170 |
| FoxTunnel_33_3 | SRX2372171 |
| FoxTunnel_33_4 | SRX2372172 |
| HessCreek_1 | SRS266168 |
| HessCreek_2 | SRS266169 |
| Kolyma_10K | SRX5008039 |
| Kolyma_800K | SRX5008037 |
| Kolyma_IC4 | SAMN03161559, SAMN03161560 |
| Kolyma_IC8 | SAMN03161561, SAMN03161562 |
| NomeCreek_3-12_90 | SAMN02380566 |
| NomeCreek_3-6_50 | SAMN02380562 |
| NomeCreek_4-12_90 | SAMN02380567 |
| NomeCreek_4-6_50 | SAMN02380563 |
| Nunavut_1 | SRX710557 |
| Nunavut_2 | SRX710561 |
| Nunavut_3 | SRX710568 |
| StordalenMire_1 | SRR7151545 |
| StordalenMire_2 | SRR7151546 |
| StordalenMire_3 | SRR7151554 |
| StordalenMire_4 | SRR7151655 |
| Svalbard_101_118 | SAMEA5229569 |
| Svalbard_118_126 | SAMEA5229570 |
| Svalbard_126_144 | SAMEA5229571 |
| Svalbard_161_181 | SAMEA5229572 |

**Table S2.** Site information, age dating, and citation resources. For samples collected and sequenced for this study, the ‘Sample Nomenclature’ column describes how samples are named and grouped. For downloaded data, the ‘Sample Age Inference’ column indicates how permafrost ages were inferred if dates were not included as part of the original study.

| Collected samples |  |  |  |
| --- | --- | --- | --- |
| Site name | **Sample Nomenclature** | **Age determination and/or citation** |  |
| Fox Permafrost Tunnel | Samples are labeled by age classification (19, 27, or 33 kyr) and core number (e.g., FoxTunnel_19_5).  One sample was collected from above the tunnel with a SIPRE corer and is called AboveFoxTunnel5_3. | Measured by Mackelprang et al., 2017 [2] |  |
| Vault Creek Permafrost Tunnel | Samples are labeled according to distance from tunnel portal. The first number in the naming scheme represents distance from the entrance, with 1 being the closest and 4 being the furthest. The second number represents core number. E.g., VaultTunnel_2_3 | Measured by Schirrmeister et al., (2016) [4] |  |
| Black Sheep Pond | Samples are labeled by core, Deep Core 2 (DC2) and Deep Core 3 (DC3), and are further differentiated by depth. | Katie Walter Anthony, personal communication |  |
| Peatball Lake | The first number represents the core number and the second represents replicate number within a core. E.g., PeatBallLake_1_1. | Measured at NOSAMS |  |
| Wadepiper Lake | The first number represents the core number and the second represents replicate number within a core. E.g., WadePiperLake_1_1. | Chris Arp, personal communication |  |
| Kaktovic | The first number represents the core number and the letter following represents a further differentiation by depth (E.g., Kaktovic_1C) | Measured at NOSAMS |  |
| Utqiagvik | Names primarily reflect sample naming conventions in the field. 154B, 159B and AB12 are from flat-centered polygons. 305 is from a low centered polygon. AB, L1, and L2 are from high-centered polygons. | Measured by Meyer et al (2010) [29] |  |
| Bol’shoy Lyakhovsky Island | Sample names reflect age: Eemian (EM), Holocene equivalent of the Eemian (HEM), and Late Pleistocene (Yed). Numbers following age designation correspond to core names published in Stapel et al (2018) [8]. 1444 corresponds to core L14-04-04, 1447 corresponds to core L14-04-7, 1452 corresponds to core L14-05-02, 14210 corresponds to core L14-02-10, and 1423 corresponds to core L14-02-03. Numbers following core designation indicate replicates within a core | Measured by Stapel et al (2018) [8] and references therein |  |
| Downloaded data | **Sample Age Inference (**If direct measurements not available) | **Age determination citation** |  |
| Atqasuk |  | Wagner et al (2017) [9] |  |
| Bonanza Creek |  | Hultman et al (2015) [10] |  |
| Eureka |  | Yergeau et al (2010) [11] |  |
| Hess Creek |  | Mackelprang et al (2011), Waldrop et al (2010) [12, 30] |  |
| Imnaviat | Nichols et al dates permafrost from similar depths (1m) in the Imnaviat Creek Watershed to be primarily from the early Holocene using radiocarbon dating. They estimate that samples from 50-100 cm are from 1320 to 1563 years and samples from 100-161cm are from 2754 to 5404 years. An estimate of 2000 years used here splits the difference | Ward et al (2017), Nichols et al (2017) [13, 31] |  |
| Kolyma | Inferred based on stratification record | Liang et al (2019), Krivushin et al (2015) [14, 32] |  |
| Svalbard | Cable et al. dates permafrost from similar depth in a nearby location as old as 7.8 kya, permafrost in this region is found to be of many different ages - 7.8 kya is their general approximation for the valley. Authors use 14C + plant fossils | Cable et al (2018), Xue et al (2019) [19, 33] |  |
| Nunavut | Dating information was not provided in the original study, but SOC 14C data was obtained from another study in the same region [34]. | Chauhan et al (2014), Ziolkowski et al (2019) [15, 34] |  |
| Stordalen Mire | Approximation based on site history. | Emerson et al (2018), Woodcroft et al (2018)[16, 17] |  |
| Nome Creek |  | Tas et al (2014) [18] |  |
| Fox Permafrost Tunnel |  | Mackelprang et al (2017) [2] |  |

**Table S3.** PERMANCOVA results table for PC1 and PC2 from PCA analysis of environmental data. df: degrees of freedom, SS: sum of squares, MS: mean sum of squares, Psuedo-F: F-value by permutation, P(perm): p values based on more than 9000 permutations, Perms: number of permutations.

| **PC1** |  |  |  |  |  |  |
| --- | --- | --- | --- | --- | --- | --- |
| Source | df | SS | MS | Pseudo-F | P(perm) | perms |
| PC1_Score | 1 | 859.7 | 859.7 | 1.3463 | 0.1493 | 9906 |
| Continent | 2 | 832.57 | 416.28 | 0.56185 | 0.5962 | 9950 |
| Region(Continent) | 1 | 1051 | 1051 | 1.4921 | 0.1594 | 9950 |
| PC1_Score x Continent | 2 | 619.31 | 309.66 | 0.72311 | 0.6965 | 9937 |
| Site(Region(Continent)) | 38 | 20792 | 547.15 | 6.361 | 0.0001 | 9842 |
| PC1_Score x Region(Continent) | 1 | 129.71 | 129.71 | 1.508 | 0.1194 | 9921 |
| PC1_Score x Site(Region(Continent)) | 22 | 4251.3 | 193.24 | 2.2466 | 0.0002 | 9873 |
| Res | 65 | 5591.1 | 86.016 |  |  |  |
| Total | 132 | 34126 |  |  |  |  |
| **PC2** |  |  |  |  |  |  |
| Source | df | SS | MS | Pseudo-F | P(perm) | perms |
| PC2_Score | 1 | 1119.6 | 1119.6 | 1.5361 | 0.1642 | 9931 |
| Continent | 2 | 756.72 | 378.36 | 0.7756 | 0.5905 | 9965 |
| Region(Continent) | 1 | 728.94 | 728.94 | 1.0027 | 0.3778 | 9934 |
| PC2_Score x Continent | 2 | 763.54 | 381.77 | 0.81155 | 0.6659 | 9911 |
| Site(Region(Continent)) | 38 | 20805 | 547.5 | 6.6487 | 0.0001 | 9880 |
| PC2_Score x Region(Continent) | 1 | 113.51 | 113.51 | 1.3784 | 0.1849 | 9910 |
| PC2_Score x Site(Region(Continent)) | 22 | 4486.4 | 203.93 | 2.4764 | 0.0001 | 9907 |
| Res | 65 | 5352.6 | 82.348 |  |  |  |
| Total | 132 | 34126 |  |  |  |  |

**Table S4**. Biophysical variation among the six major clusters. ‘n’ indicates the number of samples in a cluster. Data indicate means plus ± 1 S.D. Different superscripts indicate significant differences based on a Tukey post hoc test. P value indicates the level of significance from a one-way ANOVA.

| Cluster | 1 | 2 | 3 | 4 | 5 | 6 | p |
| --- | --- | --- | --- | --- | --- | --- | --- |
| n | 15 | 8 | 14 | 31 | 6 | 43 |  |
| Latitude (degrees N) | 72.2 ± 5.3^a^ | 69 ± 4.0^abc^ | 70.3 ± 4.4^a^ | 68.9 ± 3.4^ac^ | 64.9 ± 0.07^bc^ | 66 ± 2.9^b^ | *** |
| pH | 5.7 ± 1.0^c^ | 6.0 ± 0.7^bc^ | 6.8 ± 1.3^ab^ | 6.7 ± 1.3^b^ | 7.1 ± 0.6^ab^ | 7.4 ± 0.1^a^ | *** |
| Depth (m) | 3.6 ± 3.7^c^ | 2.4 ± 2.4^c^ | 1.1 ± 0.5^c^ | 4.3 ± 4.2^c^ | 28 ± 11.9^a^ | 12.8 ± 6.9^b^ | *** |
| %N | 1.0 ± 1.2^a^ | 0.7 ± 0.6^ab^ | 0.4 ± 0.5^b^ | 0.5 ± 0.6^ab^ | 0.1 ± 0.1^b^ | 0.2 ± 0.1^b^ | ** |
| % OC | 8.3 ± 14.5 ^a^ | 11.8 ± 14.0^a^ | 6.0 ± 7.7^ab^ | 8.3 ± 12.1^ab^ | 2.8 ± 2.9^ab^ | 2.5 ± 1.3^b^ | * |
| Age (kyr) | 20 ± 19^ab^ | 17 ± 18^ab^ | 10 ± 7^b^ | 12 ± 12^b^ | 37 ± 41^ab^ | 31 ± 28^a^ | * |

*= p < 0.05

** = p < 0.001

*** = p < 0.0001

**Table S5.** Functional genes related to substrate utilization represented in Groups A and B.

|  | Group A | | Group B | |
| --- | --- | --- | --- | --- |
|  | **A1** | **A2** | **B1** | **B2** |
| Amino acids and other nitrogenous molecules | K14155: cysteine-S-conjugate beta-lyase | K01667: tryptophanse | K01697: cystathionine beta-synthase | K01425: glutaminase |
|  | K00262: glutamate dehydrogenase (NADP+) | K01668: tyrosine phenol-lyase | K15372: taurine---2-oxoglutarate transaminase | K00274: Monoamine oxidase |
|  |  | K01372: bleomycin hydrolase (exopeptidase) | K01473: N-methylhydantoinase A | K03293: amino acid transporter, AAT family |
|  |  | K07263: zinc protease | K01474: N-methylhydantoinase B | K00263: leucine dehydrogenase |
|  |  | K01301: N-acetylated-alpha-linked acidic dipeptidase | K01488: adenosine deaminase | K03735: ethanolamine ammonia-lyase large subunit |
|  |  | K08677: kumamolisin (endopeptidase) | K03383: cyanuric acid amidohydrolase | K01721: nitrile hydratase subunit alpha |
|  |  |  | K00451: homogentisate 1,2-dioxygenase | K00822: beta-alanine—pyruvate transaminase |
|  |  |  | K00457:4-hydroxyphenylpyruvate dioxygenase | K01428: urease subunit alpha |
|  |  |  | K01712: urocanate hydratase | K11178: xanthine dehydrogenase YagS FAD-binding subunit |
|  |  |  |  | K00302: sarcosine oxidase, subunit alpha |
|  |  |  |  | K00310: alanine or glycine:cation symporter, AGCS family |
| Aromatic hydrocarbons |  | K04113: benzoyl-CoA reductase subunit B | K15512: benzoyl-CoA 2,3-epoxidase subunit B |  |
|  |  | K04108: 4-hydroxybenzoyl-CoA reductase subunit alpha. aromatic compound degradation | K00449: protocatechuate 3,4-dioxygenase, beta subunit |  |
|  |  |  | K02613: ring-1,2-phenylacetyl-CoA epoxidase subunit PaaE |  |
|  |  |  | K02609: ring-1,2-phenylacetyl-CoA epoxidase subunit PaaA |  |
| Fatty acids |  |  | K00249: acyl-CoA dehydrogenase | K00232: Acyl-CoA oxidase |
|  |  |  | K09456: putative acyl-CoA dehydrogenase | K07110: XRE family transcriptional regulator |
|  |  |  |  | K06445: Acyl-CoA dehydrogenase |
| Carbohydrates | K07457: endoglucanase | K03556: LuxR family transcriptional regulator, maltose regulon positive regulatory protein |  |  |
|  |  | K01188: beta-glucosidase |  |  |
|  |  | K00688: glycogen phosphorylase |  |  |
|  |  | K01206: alpha-L-fucosidase |  |  |
| Other | K00054: hydroxymethylglutaryl-CoA reductase |  | K00252: glutaryl-CoA dehydrogenase | K01699: propanediol dehydratase large subunit |
|  |  |  | K03336: 3D-(3,5/4)-trihydroxycyclohexane-1,2-dione acylhydrolase | K11187: xanthine dehydrogenase YagS FAD-binding subunit |
|  |  |  |  | K007795: Putative tricarboxylic transport membrane protein |
|  |  |  |  | K01569: Oxalate decarboxylase |
|  |  |  |  | K01130: arylsulfatase |

**SI References**

1. Bjella K, Tantillo T, Weale J, Lever J. Evaluation of the CRREL Permafrost Tunnel. 2008. US Army Corps of Engineers, Engineer Research and Development Center.

2. Mackelprang R, Burkert A, Haw M, Mahendrarajah T, Conaway CH, Douglas TA, et al. Microbial survival strategies in ancient permafrost: insights from metagenomics. *ISME J* 2017; **11**: 2305–2318.

3. Meyer H, Yoshikawa K, Schirrmeister L, Andreev A. The Vault Creek Tunnel (Fairbanks Region, Alaska)—a late Quaternary palaeoenvironmental permafrost record. *Ninth International Conference on Permafrost (NICOP), Fairbanks, Alaska*. 2008.

4. Schirrmeister L, Meyer H, Andreev A, Wetterich S, Kienast F, Bobrov A, et al. Late Quaternary paleoenvironmental records from the Chatanika River valley near Fairbanks (Alaska). *Quat Sci Rev* 2016; **147**: 259–278.

5. Gibbs AE, Erikson LH, Jones BM, Richmond BM, Engelstad AC. Seven decades of coastal change at barter island, alaska: exploring the importance of waves and temperature on erosion of coastal permafrost bluffs. *Remote Sensing* 2021; **13**: 4420.

6. Wu Y, Ulrich C, Kneafsey T, Lopez R, Chou C, Geller J, et al. Depth-resolved physicochemical characteristics of active layer and permafrost soils in an arctic polygonal tundra region. *J Geophys Res Biogeosci* 2018; **123**: 1366–1386.

7. Taş N, Prestat E, Wang S, Wu Y, Ulrich C, Kneafsey T, et al. Landscape topography structures the soil microbiome in arctic polygonal tundra. *Nat Commun* 2018; **9**: 777.

8. Stapel JG, Schwamborn G, Schirrmeister L, Horsfield B, Mangelsdorf K. Substrate potential of last interglacial to Holocene permafrost organic matter for future microbial greenhouse gas production. *Biogeosciences* 2018; **15**: 1969–1985.

9. Wagner R, Zona D, Oechel W, Lipson D. Microbial community structure and soil pH correspond to methane production in Arctic Alaska soils. *Environ Microbiol* 2017; **19**: 3398–3410.

10. Hultman J, Waldrop MP, Mackelprang R, David MM, McFarland J, Blazewicz SJ, et al. Multi-omics of permafrost, active layer and thermokarst bog soil microbiomes. *Nature* 2015; **521**: 208–212.

11. Yergeau E, Hogues H, Whyte LG, Greer CW. The functional potential of high Arctic permafrost revealed by metagenomic sequencing, qPCR and microarray analyses. *ISME J* 2010; **4**: 1206–1214.

12. Mackelprang R, Waldrop MP, DeAngelis KM, David MM, Chavarria KL, Blazewicz SJ, et al. Metagenomic analysis of a permafrost microbial community reveals a rapid response to thaw. *Nature* 2011; **480**: 368–371.

13. Ward CP, Nalven SG, Crump BC, Kling GW, Cory RM. Photochemical alteration of organic carbon draining permafrost soils shifts microbial metabolic pathways and stimulates respiration. *Nat Commun* 2017; **8**: 772.

14. Liang R, Lau M, Vishnivetskaya T, Lloyd KG, Wang W, Wiggins J, et al. Predominance of anaerobic, spore-forming bacteria in metabolically active microbial communities from ancient Siberian permafrost. *Appl Environ Microbiol* 2019; **85**: e00560-19.

15. Chauhan A, Layton AC, Vishnivetskaya TA, Williams D, Pfiffner SM, Rekepalli B, et al. Metagenomes from thawing low-soil-organic-carbon mineral cryosols and permafrost of the canadian high arctic. *Genome Announc* 2014; **2**: e01217-14.

16. Emerson JB, Roux S, Brum JR, Bolduc B, Woodcroft BJ, Jang HB, et al. Host-linked soil viral ecology along a permafrost thaw gradient. *Nat Microbiol* 2018; **3**: 870–880.

17. Woodcroft BJ, Singleton CM, Boyd JA, Evans PN, Emerson JB, Zayed AAF, et al. Genome-centric view of carbon processing in thawing permafrost. *Nature* 2018; **560**: 49–54.

18. Taş N, Prestat E, McFarland JW, Wickland KP, Knight R, Berhe AA, et al. Impact of fire on active layer and permafrost microbial communities and metagenomes in an upland Alaskan boreal forest. *ISME J* 2014; **8**: 1904–1919.

19. Xue Y, Jonassen I, Øvreås L, Taş N. Bacterial and archaeal metagenome-assembled genome sequences from Svalbard permafrost. *Microbiol Resour Announc* 2019; **8**: e00516-19.

20. Brown J. Radiocarbon Dating, Barrow, Alaska. *Arctic* 1965; **18**: 37–48.

21. Edgar RC. Search and clustering orders of magnitude faster than BLAST. *Bioinformatics* 2010; **26**: 2460–2461.

22. Huson DH, Beier S, Flade I, Górska A, El-Hadidi M, Mitra S, et al. MEGAN community edition - interactive exploration and analysis of large-scale microbiome sequencing data. *PLoS Comput Biol* 2016; **12**: e1004957.

23. R Core Team. R: A Language and Environment for Statistical Computing. 2021. R Foundation for Statistical Computing, Vienna, Austria.

24. McMurdie PJ, Holmes S. phyloseq: an R package for reproducible interactive analysis and graphics of microbiome census data. *PLoS One* 2013; **8**: e61217.

25. Kanehisa M, Furumichi M, Tanabe M, Sato Y, Morishima K. KEGG: new perspectives on genomes, pathways, diseases and drugs. *Nucleic Acids Res* 2017; **45**: D353–D361.

26. Buchfink B, Xie C, Huson DH. Fast and sensitive protein alignment using DIAMOND. *Nat Methods* 2015; **12**: 59–60.

27. Honaker J, King G, Blackwell M. AmeliaII: A program for missing data. *J Stat Softw* 2011; **45**: 1–47.

28. Clarke KR, Gorley RN. PRIMER v7: User Manual/Tutorial. 2006. Plymouth Maine Laboratory.

29. Meyer H, Schirrmeister L, Andreev A, Wagner D, Hubberten H-W, Yoshikawa K, et al. Lateglacial and Holocene isotopic and environmental history of northern coastal Alaska – Results from a buried ice-wedge system at Barrow. *Quat Sci Rev* 2010; **29**: 3720–3735.

30. Waldrop MP, Wickland KP, White R Iii, Berhe AA, Harden JW, Romanovsky VE. Molecular investigations into a globally important carbon pool: permafrost-protected carbon in Alaskan soils. *Glob Chang Biol* 2010; **16**: 2543–2554.

31. Nichols JE, Peteet DM, Frolking S, Karavias J. A probabilistic method of assessing carbon accumulation rate at Imnavait Creek Peatland, Arctic Long Term Ecological Research Station, Alaska. *J Quat Sci* 2017; **32**: 579–586.

32. Krivushin K, Kondrashov F, Shmakova L, Tutukina M, Petrovskaya L, Rivkina E. Two metagenomes from late pleistocene northeast siberian permafrost. *Genome Announc* 2015; **3**.

33. Cable S, Elberling B, Kroon A. Holocene permafrost history and cryostratigraphy in the High-Arctic Adventdalen Valley, central Svalbard. *Boreas* 2018; **47**: 423–442.

34. Ziolkowski L. Arctic soil microbes are not consuming ancient soil carbon: Implications for depositional environments, Canada, 2011. 2019. Arctic Data Center. doi:10.18739/A2NS0KX7H
